# Hospital survey on patient safety culture (HSOPSC): a multi-method approach for target-language instrument translation, adaptation, and validation to improve the equivalence of meaning for cross-cultural research

**DOI:** 10.1186/s12912-020-00419-9

**Published:** 2020-04-13

**Authors:** Patrick A. Palmieri, Juan M. Leyva-Moral, Doriam E. Camacho-Rodriguez, Nina Granel-Gimenez, Eric W. Ford, Kathleen M. Mathieson, Joan S. Leafman

**Affiliations:** 1 Office of the Vice Chancellor for Research, Universidad Norbert Wiener, Av. Arequipa 444, 15046 Lima, Peru; 2 College of Graduate Health Studies, A. T. Still University, 800 West Jefferson Street, Kirksville, MO 63501 USA; 3 School of Nursing, Walden University, 100 S Washington Ave, Suite 900, Minneapolis, MN 55401 USA; 4 EBHC South America: A Joanna Briggs Institute Affiliated Group, Universidad Norbert Wiener, Av. Arequipa 444, Lima, 15046 Lima, Peru; 5 Departament d’Infermeria, Facultat de Medicina, Universitat Autonoma de Barcelona, Avda. Can Domenech, Building M. Office M3/211, 08193 Bellaterra (Cerdanyola del Vallès), Barcelona, Spain; 6 Center for Health Sciences Research, Universidad María Auxiliadora, Av. Canto Bello 431, 15408 Lima, Peru; 7 EBHC South America: A Joanna Briggs Institute Affiliated Group, Universidad Norbert Wiener, Av. Arequipa 444, 15046 Lima, Peru; 8 School of Nursing, Universidad Cooperativa de Colombia, Calle 30, Santa Marta, Magdalena Colombia; 9 School of Public Health, University of Alabama at Birmingham, Ryals Public Health Building, 1665 University Blvd., Ryals 310E, Birmingham, AL 35233 USA

**Keywords:** Hospital survey on patient safety culture, Patient safety, Organizational culture, Instrument translation, Instrument adaptation, Instrument validation, Cross cultural research, Agency for Healthcare Research and Quality, Spanish, Peru

## Abstract

**Background:**

The Hospital Survey on Patient Safety Culture (HSOPSC) is widely utilized in multiple languages across the world. Despite culture and language variations, research studies from Latin America use the Spanish language HSOPSC validated for Spain and the United States. Yet, these studies fail to report the translation method, cultural adaptation process, and the equivalence assessment strategy. As such, the psychometric properties of the HSOPSC are not well demonstrated for cross-cultural research in Latin America, including Peru. The purpose of this study was to develop a target-language HSOPSC for cross-cultural research in Peru that asks the same questions, in the same manner, with the same intended meaning, as the source instrument.

**Methods:**

This study used a mixed-methods approach adapted from the translation guideline recommended by Agency for Healthcare Research and Quality. The 3-phase, 7-step process incorporated translation techniques, pilot testing, cognitive interviews, clinical participant review, and subject matter expert evaluation.

**Results:**

The instrument was translated and evaluated in 3 rounds of cognitive interview (CI). There were 37 problem items identified in round 1 (14 clarity, 12 cultural, 11 mixed); and resolved to 4 problems by round 3. The pilot-testing language clarity inter-rater reliability was S-CVI/Avg = 0.97 and S-CVI/UA = 0.86; and S-CVI/Avg = 0.96 and S-CVI/UA = 0.83 for cultural relevance. Subject matter expert agreement in matching items to the correct dimensions was substantially equivalent (Kappa = 0.72). Only 1 of 12 dimensions had a low Kappa (0.39), borderline fair to moderate. The remaining dimensions performed well (7 = almost perfect, 2 = substantial, and 2 = moderate).

**Conclusions:**

The HSOPSC instrument developed for Peru was markedly different from the other Spanish-language versions. The resulting items were equivalent in meaning to the source, despite the new language and different cultural context. The analysis identified negatively worded items were problematic for target-language translation. With the limited literature about negatively worded items in the context of cross-cultural research, further research is necessary to evaluate this finding and the recommendation to include negatively worded items in instruments. This study demonstrates cross-cultural research with translated instruments should adhere to established guidelines, with cognitive interviews, based on evidence-based strategies.

## Background

As many as 16% of hospitalized patients experience a medial error during their health encounter [1]. Hospital culture and patient safety are interrelated as organizational failures and system driven errors contribute to the unintended events that produce poor quality outcomes [2]. Prominent international agencies, such as the World Health Organization (WHO) and the United States Agency for Healthcare Research and Quality (AHRQ), recommend hospitals address this problem by improving their organizational cultures [3]. As such, health services researchers are interested in the intersection of organizational culture with patient safety [4, 5]. Safety culture can be defined as the overarching but emergent healthcare property where professional attitudes and work climates result in the degree of system reliability and resilience to adverse outcomes [6].

Clinical quality and patient safety outcomes are linked to the organizational culture dimensions that can be measured with safety culture instruments for hospitals [7]. Safety culture measures are correlated with employee performance (e.g. safety behavior), process and system errors, and accident rates across industries [8] and cultures [9] with similar results from the health sector [10]. For example, researchers using the Hospital Survey on Patient Safety Culture (HSOPSC) have reported strong correlations between safety culture, adverse event frequency, and patient outcomes [11, 12]. For these reasons, the measurement of safety culture has become the prerequisite for continuous quality improvement efforts to provide leaders with the essential feedback that stimulates organizational improvement [13–15].

In developing countries basic hospital safety indicators are largely incomplete or unavailable [16–18]. The sparse available data indicates adverse event prevalence in South American countries, including Peru [19–21], is much higher than developed countries [22, 23]. Patient safety is an emerging focus in the Peruvian health sector, as demonstrated by the lack of basic safety programs, processes, and practices [24]. The HSOPSC is a validated tool to measure the effectiveness of work environment and organizational process associated with preventing the types of errors linked to consequential adverse events [25]. When administered yearly, the HSOPSC can provide leaders with a proxy measurement for the effectiveness of their quality improvement efforts focused on achieving patient safety [13].

Early instruments to measure safety culture were developed in the late 1980’s and into early 1990’s [14] but did not gain popularity due to poorly performing psychometric properties [15]. In a seminal review of contemporary instruments [26], multiple safety culture instruments were identified but varied considerably in the general characteristics, dimensions covered, and psychometric properties. Furthermore, the items, and associated dimensions, were not derived from a theoretically constructed framework [13]. In response to the need for a standard instrument to measure safety culture, the AHRQ commissioned the development of a psychometrically valid and reliable instrument [27–29], called the HSOPSC.

The HSOPSC, developed in English [28] for use in American hospitals, has demonstrated excellent psychometric properties [27]. The instrument has been disseminated to other countries, in multiple languages [8, 24, 30–36]; however, published studies generally neglect to report the instrument translation method and validation data. When provided, the information is either limited or poor quality such as reporting a simple post hoc psychometric analysis. In this regard, a Spanish language version of the instrument is available for use with Spanish speaking health workers in the United States [37]. This version has also been used in Mexico [38, 39], Colombia [40], and most recently Peru [24]. Also, a different Spanish-language version of the instrument [41] was developed for use in Spain [42]. However, studies applying either of these Spanish language instruments for cross-culture research do not report the validation method and provide limited psychometric data about reliability. According to the basic recommended translation strategies published by the AHRQ [43], there is no validated Spanish language version of the HSOPSC reported in the literature for cross-cultural research in Peru, as well as in other countries in Latin America. As the Spanish language differs between countries and the meaning of words differs across cultures, a validated Spanish language version of the HSOPSC needs to be developed for cross-cultural research in Peru, that confirms construct applicability and survey item integrity [44, 45].

Researchers working with Spanish speaking populations [46, 47] have reported the traditional forward-reverse translation technique does not result in a valid target-language instrument [48, 49]. However, the item meanings, dimension integrity, and construct validity need to remain constant regardless of the language across cultures [50], with an attention to eliminating bias and maximizing equivalence [51]. In order to study patient safety culture in a global perspective, the HSOPC needs to be translated from the source language (original language) into the target language (translated language) without losing the meaning [52] and context of the items and associated scales and/or dimensions during the translation process [51]. As such, the purpose of this study was to test a mixed method approach for target-language instrument translation to produce a valid HSOPSC translation for cross-cultural research in Peru.

Developing a psychometrically sound target-language instrument for cross-cultural research from an English-language source, originally validated for use in the United States, is not straightforward [53]. In cross-cultural research, the assumption that all instruments will automatically be equivalent across groups does not hold [54]. However, instruments that are products of their local environments at the time they were developed are likely to be more reliable [55]. Yet, researchers consistently fail to describe how the HSOPSC was translated and validated [56] for cross-cultural comparability and compatibility [57]. For cross-cultural research, the technical and semantic equivalence and cultural relevance of each item needs to be evaluated prior to data collection [58–60]. Without a sufficient pre-data collection evaluation to ensure the correct representation of the instrument [60], the resulting factor analyses post-data collection will be flawed and less rigorous [56].

## Methods

Despite the increased adaptation of English language instruments for cross-cultural research, there is not consensus about the gold standard method for instrument translation and validation, including cultural adaptation [56, 61, 62]. However, proper cross-cultural research with instrument translation generally requires multiple qualitative and quantitative methods and techniques [55, 56, 61, 63], including feedback questionnaires, pilot testing, expert panels, and cognitive interviews [63–65]. As such, using an iterative mixed method approach to translate instruments [46], with cognitive interviews [57, 66] ensures the resulting instrument will have equivalence [51]; one that asks the same questions, in the same manner, with the same intended meaning, as the source instrument.

With globalization in the context of cross-cultural research, evidence-based methods are required to produce equivalent target-language instrument translations from the sources [52, 67]. As such, this is a mixed-method approach adapted from the translation guidelines recommended by AHRQ [43, 68, 69] with reference to other best practices and strategies [63, 70–72]. The approach adhered to the adapted version of the equivalence criteria for cross-cultural research with instruments [50, 55, 63, 73], see Table 1. The AHRQ provided written permission by email (CRM:00350304) to use the HSOPSC in English and Spanish for this research project. In addition, permission was granted to publish the resulting instrument from this study as well as the existing Spanish and English HSOPSC instruments.

**Table 1:** Equivalence Criteria for Cross-Cultural Research with Instruments

Criteria	Definition	Process
Content Equivalence	The content of each item of the instrument is relevant to the phenomena of each culture being studied.	— Research Team Experts. — Clinical Practice Experts. — Subject Matter Experts. — Content validity index score. — Annotated survey dimension document.
Semantic Equivalence	The meaning of each item is the same in each culture after translation into the language and idiom (written or oral) of each culture.	— Translation guide from AHRQ. — Qualified / experienced translators. — Forward- and reverse-translation. — Pilot test (cultural relevance & readability). — Confirmation of translation: Cognitive interviews and expert reviews.
Technical Equivalence	The method of assessment is comparable in each culture with respect to the data that it yields.	— Translation guide from AHRQ — Experienced translators. — Subject Matter Experts. — Pilot test (evaluation scores). — Cognitive interviews.
Criterion Equivalence	The interpretation of the measurement of the variable remains the same when compared with the norm for each culture studied.	— Research Team Experts. — Subject Matter Experts. — Pilot test (evaluation scores). — Cognitive interviews.
Conceptual Equivalence	The instrument is measuring the same theoretical construct in each culture.	— Translation guide from AHRQ. — Qualified / experienced translators. — Forward- and reverse-translation. — Item to dimension selection/match. — Dual scoring process with content validity index and pilot test..

Adapted from Squires et al. [63], and Flaherty et al. [50]

### Data collection

Per the recommendations for cross-cultural instrument research where the source country, culture, and language are different than the target [72], the data collection process required three phases, including: Instrument translation, Cultural-adaptation, content validation and equivalence [47, 72]. These phases included a forward and reverse translation, cognitive interviews, targeted participant review, structured pilot test, content validation, and expert evaluation of equivalence. The work completed in each of the phases are described next.

### Participants

Phase 1, the translation process, was completed by three bilingual (English/Spanish) professionals, with at least an undergraduate degree in linguistics and extensive experience in translation and interpretation. Phase 2 included nine participants (2 nurses and 7 physicians), called clinical practice experts (CPEs), purposefully selected from licensed health professionals in Peru with current hospital work experience, fluency in Spanish, and advanced English skills as self-reported and observed by the principal researcher during a brief interview. Phase 3, included 7 participants (4 nurses, 2 physicians, 1 pharmacist), called subject matter experts (SMEs), purposefully selected as recommended by Grant and Davis [74], intermediate to advanced English was required. All participants verbally consented to participate in this study approved by the A.T. Still University Institutional Review Board (Protocol #01146).

### Instrument HSOPSC

The HSOPSC is a 42-item instrument grouped into 12 composite dimensions and nine non-dimensional items, including two safety assessment items and seven demographic items [69]. Each dimension is represented by three to five items measured with a 5-point Likert scale to ascertain agreement (strongly disagree to strongly agree) or frequency (never to always). The instrument includes 59.52% positively worded items and 48% negatively worded items. The outcome measures are the two single-item responses inquiring about the number of events reported within the past 12 months and the overall patient safety score (excellent to failing). For the reported error questions, errors are defined as any type, without regard to harm.

### Instrument translation – phase 1

#### Step 1: instrument review and translator selection

The source American English version of the HSOPC was reviewed by two bilingual healthcare experts with the primary investigator to establish the content equivalence, or the relevance and sensitivity [50, 75]. Although some challenging terms and phrases were noted in items, through discussion, the experts determined the instrument content was generally adaptable for translation and implementation in Peru. As such, the instrument was sent for translation without changes [74].

#### Step 2: forward translations with synthesis

The instrument was independently forward-translated from American English to Peruvian Spanish by two translators [76, 77]. Then, the two versions were synthesized and consolidated into a single instrument [75] by the two healthcare experts and the primary investigator, all knowledgeable about the instrument properties and scientific foundation. The translators were involved in this process specifically for grammar and language clarifications.

#### Step 3: reverse translation with reconciliation

The goal of the reverse-translation process was to establish a conceptual rather than a literal “word-by-word” meaning [78]. An independent bilingual translator reverse-translated the synthesized instrument from Peruvian Spanish to American English. Then, the reverse-translated instrument was examined, in comparison to the original instrument, by the two healthcare experts and the primary investigator. Although discrete differences were identified in the reverse-translation, these were primarily related to nearly equivalent verb selections. There were five instances where phrases required clarification to achieve the same meaning in Spanish as expressed in English. However, these clarifications were noted for discussion during the cognitive interview phase. Again, the translator provided clarifications specific to grammar and language.

### Cultural-adaptation – phase 2

Although a pilot test with a bilingual version of the instrument has been recommended to determine the equivalence between the newly translated and the original instruments [79], there were not enough bilingual participants readily accessible for this study. Alternatively, this study used a pretest to review and refine the instrument through a series of three rounds. The cognitive interviews followed each pretest to assesses the four stages of the participant engagement in completing the instrument, including: Comprehension, retrieval, judgement, and responding [80]. The procedure is described below.

#### Step 4. Pre-test

Nine CPEs engaged in the pilot testing with three CPE per round across three sequential rounds. First, the CPEs completed the instrument without interruption. Then, the CPEs were asked for their general perspective about the instrument. Finally, all CPEs were asked to complete the new instrument and for a final assessment for item clarity and cultural equivalence. The assessment required each CPE to respond to each item and then rank the item on a five-point Likert scale for language clarity (5 – completely readable and understandable, 4 – mostly readable and understandable, 3 – readable and understandable, 2 – somewhat readable and somewhat understandable; and 1 – not readable and understandable) and cultural equivalence (5 – completely culturally relevant, 4 – mostly culturally relevant, 3 – culturally relevant, 2 – somewhat culturally relevant, 1 – not culturally relevant). This coding schematic was necessary to identify the aggregate level of item clarity and cultural relevance across participants. The assessment data was entered into an Excel database for the analysis. The item specific scores guided the primary focus of the first round of cognitive interviews; scores less than four were considered opportunities for improvement.

#### Step 5. Cognitive interviews

Once the data entry was completed, the primary investigator conducted a cognitive interview with each CPE [69, 81], through a structured but open-ended discussion, to understand how participants read, comprehended, and responded to each item [57]. A cognitive interview script guided the content probes for additional evaluation of items scoring three or less in the first round and items scoring four or less in the subsequent rounds. This probative process was constructed to identify translation deficiencies by asking CPEs to describe their understanding of the item in Spanish and then again after considering the original question in English. Furthermore, the other two Spanish HSOPSC versions, United States [37] and Spain [41], were incorporated into the process as resources to review, discuss, analyze, and refine problematic items. The CPEs were also asked to identify items with unfamiliar or inappropriate grammar and syntax. Cognitive interview data was thematically coded to identify problematic items [81]. Notes were collected and compiled throughout the process.

#### Step 6. Research team review with item revision

The data from the pilot testing for each round was individually and aggregately reviewed by the research team with the primary focus on improving items with ratings less than four. The notes from the cognitive interviews were also referenced when reviewing each item. Revisions for problems with items was completed to improve the item performance in the next round. The translators from the forward- and reverse-translation were consulted with specific questions about word selections and phrase clarification. All revisions were noted in the Excel spreadsheet to provide a record of the progressive evolution of each item across rounds. Each round was conducted sequentially and distinctly but all items were compared between and across rounds.

### Content validation and equivalence evaluation – phase 3

#### Step 7a. Subject matter expert evaluation of content validity

The SMEs were provided the final instrument for review with a content validity questionnaire [82–87]. Each item in the preliminary final instrument was rated on a 4 point-Likert scale (1-irrelevant, 2-little relevance; 3-relevant; and 4-extremely relevant). In addition, the item evaluation used during the pilot testing for language clarity (readability/understandability) and cultural relevance (context) was incorporated with dichotomous acceptable or unacceptable scoring. Finally, there was a space for open ended comments for each item and at the end of the questionnaire. The content validity index (CVI) and the item evaluation for language and context was calculated per item [83]. For analysis, the rankings were merged into two categories as items scored one and two were considered irrelevant and items scored three and four were considered relevant [82]. Then, the CVI was calculated individually for each item (proportion of experts rating the item as relevant). The items were then calculated as part of the twelve dimensions, as well as for the total instrument as the mean CVI [86]. A significant CVI for this process was set, a priori, at 0.70 or greater [82, 85, 86].

#### Step 7b. Subject matter expert evaluation of equivalence

The SMEs were provided with the final items from the instrument and asked to indicate which items are associated with which dimensions. Each expert was required to select only one of the twelve dimensions for each item, but they were also asked to indicate a secondary dimension, with a short rational, in the case they were unable to easily decide the dimension for the item. Through this process, the equivalence can be established. The Kappa value was calculated for the SME agreement in identifying each item with the correct dimension. The minimally acceptable Kappa value was set to 0.40 [88–90] as values below this point are not considered robust [91, 92]. According to the scale of Landis & Koch [93], the strength of the Kappa coefficients were defined in groupings (0.01–0.20 slight; 0.21–0.40 fair; 0.41–0.60 moderate; 0.61–0.80 substantial; 0.81–0.99 almost perfect, and 1.00 perfect).

## Results

### Sample characteristics

The pre-tests with cognitive interviews were completed with nine Peruvian health care professionals (6 males and 3 females) across three rounds with three different participants per round. The mean participant age was 44.6 years (± 6.7) and the mean experience level was 16.7 (± 6.5). In the previous five years, all participants worked in private hospitals (known as ‘*clínicas’* in Peru), six also worked in social security hospitals (Social Health Insurance of Peru), and five also worked in public hospitals (Ministry of Health of Peru). All participants reported working between two to four different jobs, either part-time, full-time, and/or contracted. All participants were advanced to fluent in English (6 advanced, and 3 fluent) and all participants were fluent in Peruvian Spanish (native language). Table 2 summarizes the demographic and professional characteristics.

**Table 2:** Participant Characteristics for Cognitive Interviews (*N* = 9)

Mean Age (SD)	44.6 (6.7)	
Male	6 (55.6)	
Health Professions Specialty		
Internal medicine	3 (33.3)	
Gynecology medicine	2 (22.2)	
Anesthesiology	1 (11.1)	
Pediatric medicine	1 (11.1)	
Intensive care nurse	1 (11.1)	
Medical-surgical nurse	1 (11.1)	
Mean Years of Experience (SD)	16.7 (6.5)	
Hospital Setting ^a^		
Clinica	9 (100)	
EsSalud	6 (66.7)	
MINSA	5 (55.7)	
English Comprehension		
Advanced	6 (66.7)	
Fluent	3 (33.3)	

Note: Values are expressed as *N* (%) unless otherwise noted

^a^ Participants could indicate experience in more than one hospital setting

### Pilot testing and cognitive interviews

The pilot testing of the instrument required on average 18 min (range 15 to 22 min), while the cognitive interviews required on average 50 min (range 40 min to 85 min). From the item evaluations, the participants identified multiple problem items: Round 1 there were 37 problem items (14 unique to language clarity, 12 specific to cultural, and 11 mixed); Round 2 there were 14 problematic items (6 specific to language clarity, 5 specific to cultural, and 3 mixed); and Round 3 there were four problematic items (2 specific to language clarity, 2 specific to cultural, and 0 mixed). The type, frequency, and the mix of issues improved progressively across rounds. In the aggregate analysis for each round, the language clarity issues were the most common with the lowest point percentage (Round 1 = 77.4%) followed by cultural relevance (Round 1 = 81.3%). The analysis evidenced although the initial translation was adequate from a linguistics perspective, there were opportunities to improve the clarity and relevance from the targeted population perspective [94, 95]. With focused item revisions between rounds, the cumulative item measurements for language clarity and cultural relevance improved by 10% in round 2 (79.4 to 89.2%) and improved 5% from round 2 to round 3 (89.2 to 94.5%). From the interviews, participants identified problems understanding negatively worded items. However, the participant feedback resulted in improvements to all negative items for the final version of the instrument. Table 3 summarizes the aggregate item analysis for all scores.

**Table 3:** Participant Item Rating (Aggregate Raw Score by Measure Type by Round)

Testing Round	Language Clarity^a^	Cultural Relevance^a^	Total^b^
Round 1	201.3 (77.4%)	211.3 (81.3%)	412.7 (79.4%)
Round 2	227.3 (87.4%)	236.7 (91.0%)	464.0 (89.2%)
Round 3	243.0 (93.5%)	248.3 (95.5%)	491.3 (94.5%)

^a^ Score calculation 51 items, 1–5 points per item, % is X points / 255 total

^b^ Score calculation 51 items, 1–5 points per item for each score, % is X points / 510 total

Overall, the cognitive interviews identified translation issues across rounds. For example, Round 1 had 33 problems (language clarity 42.5%, cultural 33.3%, and general design 24.2%) but this number decreased to only four problems in Round 3 (50.0, 25.0, and 25.0% respectively). Table 4 provides a summary of the item issues related to translation, context, and design. The remaining four issues identified in Round 3 improved from Round 1 as multiple participants initially rated these items as a one or two and, in the end, only a single participant rated one item as a three. The participant response for the sole issue in Round 3 was reportedly based on a negative impression about the item versus a lack of understanding or concern about cultural relevance. At the conclusion of the three-phase process, the expert panel believed each item was acceptable in terms of readability, comprehension, and cultural relevance. Table 5 presents examples of the language clarity issues identified by the participants. Regarding the cultural issues, the predominant problems were related to defining definitions of concepts or seemingly general knowledge across cultures, with two specific causes: 1) Organizational constructs that differed between the United States and Peru; and 2) References to health system constructs in the United States that are not present or not well known to professionals in Peru. Table 6 presents examples of the cultural relevance issues identified by the participants.

**Table 4:** Problems Identified from Cognitive Interviews (Number and Percent)

Round ^a^	Language Clarity	Cultural Relevance	Design	Total
Round 1	14 (42.5%)	11 (33.3%)	8 (24.2%)	33 (100%)
Round 2	7 (43.8%)	5 (31.2%)	4 (25.0%)	16 (100%)
Round 3	2 (50.0%)	1 (25.0%)	1 (25.0%)	4 (100%)

^a^ Number reported per identified problem per item regardless of frequency per item

**Table 5:** Examples of Language Clarity Issues Identified from Cognitive Interviews

Translation issue	Item content	Illustrative example
Spanish words do not convey intended construct	*Problem:* Crisis mode or “modo de crisis”	*Description of issue:* “Crisis mode” translates to “modo de crisis” en other Spanish speaking countries. The concept exists in Peru but translates differently. *English question:* We work in “crisis mode” trying to do too much, too quickly. *Initial translation:* Trabajamos en “modo de crisis”, tratando de hacer demasiado y muy rápidamente. *Final translation:* Trabajamos bajo presión intentando realizar demasiadas cosas muy rápidamente.
	*Problem:* Perden or to lose for “falls through the cracks”	*Description:* “Falls between the cracks” translates into Spanish as “perden” or to lose things which is slightly different in meaning for participants. *English question:* Things “fall between the cracks” when transferring patients from one unit to another. *Initial translation:* Las cosas se pierden cuando los pacientes son transferidos de una unidad a otra. *Final translation:* La información y/o objetos de los pacientes se pierde cuando éstos se transfieren de una unidad a otra.
Spanish words are unfamiliar or have different meanings in some cultures or different nationalities	*Problem:* Importante información for specific information important to patient safety	*Description of issue:* Information is a generic word in Spanish and the participants wanted further clarification about what information (e.g. documents or forms) is important. *English question:* Important patient care information is often lost during shift changes. *Initial translation:* Con frecuencia se pierde importante información sobre el cuidado del paciente durante los cambios de turno. *Final translation:* Se pierde a menudo información importante de cuidado de pacientes durante cambios de turno.
	*Problem:* Linking the meaning of mistakes to chance through the phrase “por casualidad”	*Description of issue:* The phrase for chance in Peru exists but linking this to mistakes that occur in the hospital requires careful word order arrangement. *English question:* It is just by chance that more serious mistakes don’t happen around here. *Initial translation:* Aquí no suceden errores más serios sólo por casualidad. *Final translation:* Es sólo por casualidad que errores más serios no suceden aquí.

**Table 6:** Examples of Cultural Relevance Issues Identified from Cognitive Interviews

Cultural issue	Item content	Illustrative example
Definition of concepts or knowledge differ across cultures or nationalities	*Problem:* Different healthcare constructs in the provision of care between the United States and Peru	*Description of issue:* Temporary staff and professionals are not frequently utilized in Peruvian healthcare facilities. *English Question:* We use more agency/temporary staff than is best for patient care. *Initial translation:* Usamos más personal temporal o de agencia de lo conveniente para el cuidado del paciente. *Final translation:* Usamos más personal de reemplazo o temporal de lo que es mejor para el cuidado del paciente.
	*Problem:* Reference to errors and recording in personnel files	*Description of issue:* The concept of blame and recording of errors in a personal file is not clear in Peru. *English question:* Staff worry that mistakes they make are kept in their personnel file. *Initial translation:* Al personal le preocupaba que los errores cometidos permanezcan en sus expedientes personales. *Final translation:* Cuando se comete un error, el personal teme que eso quede en su expediente.

### Content validity and equivalence

From the seven SME invited to participate in the item content review, only six completed the entire process. After reviewing the items (*N* = 42) for clarity, the S-CVI/Avg (scale-level content validity index, average) was 0.97 and the S-CVI/UA (scale-level content validity index, universal agreement) was 0.86 with a total item agreement of 36 of 42 items (4 items at 0.83, and 2 items at 0.67) and for cultural relevance, the S-CVI/Avg was 0.96 and the S-CVI/UA was 0.83 with a total item agreement of 35 items (5 items = 0.83, 1 item = 0.67, and 1 item = 0.50). For the equivalence of the items to the dimension for the entire instrument, the Kappa value was 0.72, or substantially equivalent. In terms of the individual dimensions, only one of the twelve dimensions (dimension 2) had a Kappa value of 0.39, which is at the borderline of moderate and fair. The remaining dimensions performed well (7 = almost perfect to perfect, 2 = substantial, and 2 = moderate).

When aggregating the HSOPSC items, then averaging the readability and cultural relevance scores for the three participants for Round 1, there were 15 items (10 for readability and 5 for cultural relevance) which scored < 3.00. For these items, 87% were negatively worded questions (readability 8 of the 10 items and cultural relevance all 5 items). In Round 2, of the 15 identified issues (those < 3.00 in Round 1), only one negatively worded item remained problematic (average score = 2.67). In Round 3, the only two items receiving an individual score less than four on either readability or cultural by participants were negatively worded. Finally, cognitive analysis revealed three additional items, recommended by participants, which should be added to the instrument. These questions are specific to the reporting systems for errors and adverse events as the participants realized these systems are either not present or not widely utilized. This represents a cultural difference in the construction of the instrument that needs to be considered for interpretation with the single measurement item. Table 7 presents the additional questions identified by the participants, but not included in the final instrument.

**Table 7:** Additional Questions Recommended by Participants

Rational for recommendation	Proposed question
Question specific to the presence of a adverse event reporting system in the facility. There is another question specific to the reporting of adverse events but not one about the presence of the system.	En su hospital funciona el Sistema de Notificación Hospitalario de eventos adversos.
Question specific to the frequency the person has reported an adverse event.	Cuando se reporta un evento ¿con qué frecuencia es analizado?
Question specific to the rating of the patient safety in the hospital or clinic. There is another question specific to rating the area or unit.	Por favor, dele a su HOSPITAL de trabajo una calificación promedio de acuerdo al grado de seguridad del paciente.

In addition to the translational, cultural, and general design issues, some navigational issues were identified during the cognitive interviews. For example, two of the three participants in Round 3 indicated they were confused about when to turn the instrument pages to answer additional items. Similarly, participants found it difficult to determine when they completed the entire instrument. Another participant indicated the choices for professions were not listed in a sensible “ranked” order and yet another participant indicated the demarcation for professional experience and years working in a facility were not clear. Finally, one participant in Round 2 and two participants in Round 3 commented that breaking item sections between pages was distracting and seemed to disrupt the instrument flow.

## Discussion

Similar to Levin et al. [47], the item analysis revealed two types of translational issues: 1) Words which when translated from English to Spanish did not convey similar constructs; and 2) Phrases or specific words which when translated from English to Spanish were not familiar to the participants as they had different meanings across cultures and/or national borders. Through the cognitive interview process; however, the participants refined these issues into four distinct domains, including: 1) translation (“reads wrong”); 2) culture relevance (“don’t understand”); 3) general instrument design (“looks strange”); and 4) navigational issues with completing instrument (“where do I go” or “what do I do” next). The fourth category has not been reported in the literature but describes some problems associated with the format and flow of the instrument. Finally, the participants realized one item asked about patient safety outcomes. As such, they all recommended additional items focused on the types and quantities of adverse errors observed in practice or personally committed.

The general design issues identified during the cognitive probing process, can be classified into four categories: 1) Formal structures and process; 2) Physician work environment; 3) Professional domains; and 4) Terminology for the public and private systems. Although the first three categories were improved with relatively minor item adjustments, the final category necessitated an instrument design with words to satisfy the distinct vocabulary of private hospitals (called *clínicas*) and public hospitals (*called* hospitales). As such, the final instrument included specific words in combination such as “hospital/clinica” and “area/unidad” to satisfy the differences in vocabulary additional items”. Table 8 presents examples of the four categories of general design issues.

**Table 8:** Examples of General Design Issues Identified from Cognitive Interviews

General design problem	Illustrative example
Health system differences between countries	*Description of issue:* In Peru, there is not a formal adverse event and reporting system at every facility. As such, the concept is unfamiliar to many participants. *Revision:* Addition of a question specific to whether or not the participant is aware of the presence of a formal error reporting system at their respective facility.
Work environment for physicians is different	*Description of issue*: In Peru, most physicians work at more than one facility. Also, they are usually employed full-time by the public health system (or semi-public) and then work as contractors in the private health system. *Revision:* Incorporated a question to capture the number of hours worked at all health facilities in addition to their primary work facility in Peru.
Professional domains are different or absent	*Description of issue:* There are many professions in the United States and in Peru which are different. For example, registered nurses are present in both places but respiratory therapists do not exist in Peru. *Revision:* Reconstruct the questions specific to professional roles and positions in facilities in Peru
Multiple concepts are different between the public and private health systems	*Description of issue:* Within Peru, there are differences between the reference terms for areas, departments, and even facilities. *Revision:* For example, the public sector term for an acute care facility is “hospital” while the private sector term is “clinica.” *Additional step:* Incorporate changes by crafting two instruments: 1) Instrument specific for public facilities; and 2) Instrument for private facilities but only with changes to distinct descriptions and terminology but without changes to the item meaning.
	*Description of issue:* Within Peru, there are differences in job titles and descriptions between the public and the private health facilities. *Revision:* For public system, use the appropriate terms and for the private system use the specific terms. *Additional step:* Incorporate changes throughout the instrument for public facilities and then throughout the instrument for private facilities.

Across the cognitive interviews, participants consistently identified similar issues with the identified problematic items. For example, in Round 2 about 82% of the items rated by the participants as a three or less, at least two of the three participants identified the same or similar issue. Also, there were multiple items where the identification agreement highlighted items that the traditional forward- and reverse-translation process would have missed. For example, a cultural issue specific to asking nurses and physicians about work hours in their primary facility was captured by cognitive interviews. In Peru, nurses and physicians routinely work enough hours at two or three facilities to be equivalent to two full-time jobs in the United States. Often, professionals are employed full-time by the public health system (or semi-public) and then work full-time in the private health system. As such, the four participants suggested we needed to incorporate an additional question to capture the total number of hours worked at all facilities in addition to their primary work facility.

Most items translated were etic [52], or concepts that were universally transferable. These items had very good clarity and substantial cultural relevance, without or with quite minor modification. Then, there were some emic concepts that needed to be addressed by the research team. These are the items reflecting culturally specific concepts in meaning, such as ideas and behaviors, in the source language [52], but prove to be inequivalent with only translation [96]. For example, an English worded item, “We work in ‘crisis mode’ trying to do too much, too quickly” translated easily but the language did not capture the meaning of the item. Although ‘crisis mode’ translates easily to ‘modo de crisis’ in Spanish speaking countries, the concept translates different in many countries such as Peru. As such, the initial translation included the phrase ‘modo de crisis’ (Trabajamos en “modo de crisis”, tratando de hacer demasiado y muy rápidamente) required significant modification that did not include the phrase (Trabajamos bajo presión intentando realizar demasiadas cosas muy rápidamente).

Another example of a seemingly discrete issue was discovered with the word ‘chance’. Although ‘chance’ exists in Spanish, when ‘chance’ is linked in a phrase with the word ‘mistake’ the participants had difficulty understanding the context of the item. In English, the item is “It is just by chance that more serious mistakes don’t happen around here” and the initial translation was “Aquí no suceden errores más serios sólo por casualidad”. By rearranging the word order and using an underline to emphasis the ‘sólo por casualidad’ the final translation was “Es sólo por casualidad que errores más serios no suceden aquí”. In this case, the item score improved significantly from an average of a 2.3 to a 4.7 in the next round and the cognitive interviews generated no additional concerns.

With the final item analysis for grammar, syntax, and other issues, we discovered negatively worded items performed poorly, required multiple conversations during cognitive interviews, and generated disagreement between experts in panel discussions. With using only positively worded questions there is higher risk for acquiescence bias [97–99]; however, the literature in this regard is primarily from English language countries and cultures. Similar to our finding about negatively worded questions, Solis-Salazar [100] reported combining positive and negative items can seriously damages the internal consistency of dimensions or subscales in Spanish language instruments.

Finally, this study has three limitations. First, the cognitive interviews required health professionals with advance to almost native levels of English comprehension. As such, these professionals might have more knowledge about safety culture than those without the same level of comprehension. In addition, the English language requirement narrowed the number of varied professionals to participate in the cognitive interviews. Second, this study assumes the etic approach to cross-cultural research. In this regard, the theoretical assumptions and operational constructs specific to safety culture were assumed to be translatable to Peru. However, the study method was a strength in providing evidence the language used to describe the assumptions and construct was transferable. Third, and last, the translators from the forward and reverse translation process were utilized as language consultants for the expert deliberations. In this regard, with justification, the “purity” of the forward and reverse translation was not maintained. A side-by-side instrument comparative each item, by dimensions, is provided in the Supplemental Table 1 for the AHRQ English and Spanish versions, the Spanish Version for Spain, and the instrument produced from this study.

## Conclusion

This is the first study to report the application of the AHRQ recommended target-language translation process, with the optional cognitive interviews, as an effective method for cross-cultural research. Incorporating cognitive interviews into a rigorous translation method resulted in an equivalent target-language HSOPSC for cross-cultural research. The feedback from the cognitive interviews contributed to substantially improved items as well as systematically validated the resulting Spanish version of the HSOPSC for the Peruvian context. By pilot testing each item with a numerical rating provided a comparison within and between rounds, the resulting evaluation was robust in assessing the clarity and cultural applicability, and provided a composite quality score. The sequential rounds revealed improved item ratings for readability and cultural relevance and the cognitive interviews resulted in successive reductions in item revisions. Furthermore, our analysis identified negatively worded questions are problematic for target-language translations from American English to Peruvian Spanish. The inclusion of a content validity measurement provided additional evidence about semantic equivalence. Finally, this is the first HSOPSC instrument translation, adaptation, and validation study reported in the literature for Latin America and appears to be the only such study for all studies undertaking the translation of the HSOPSC.

## Supplementary information

**Additional file 1 Table S1.** Item Comparison Across Instruments: Original English and Spanish Versions

## Data Availability

The datasets used and/or analyzed during the current study available from the corresponding author on reasonable request.

## Abbreviations

AHRQ: Agency for Healthcare Research and Quality
CPE: Clinical practice expert
CI: Cognitive interview
CVI: Content validity index
HSOPSC: Hospital Survey on Patient Safety Culture
I-CVI: Item-CVI
S-CVI: Scale-level content validity index
S-CVI/Avg: Scale-level content validity index, average
S-CVI/UA: Scale-level content validity index, universal agreement
SME: Subject matter experts

## References

[1] Jha AK, Prasopa-Plaizier N, Larizgoitia I, Bates DW (2010). Research priority setting working group of the WHOWAfPS: patient safety research: an overview of the global evidence. Qual Saf Health Care.

[2] Clarke SG (2006). The relationship between safety climate and safety performance: a meta analytic review. J Occup Health Psychol.

[3] Kohn LT, Corrigan JM, Donaldson MS (2000). To err is human: building a safer health system.

[4] Gershon RR, Karkashian CD, Grosch JW, Murphy LR, Escamilla-Cejudo A, Flanagan PA, Bernacki E, Kasting C, Martin L (2000). Hospital safety climate and its relationship with safe work practices and workplace exposure incidents. Am J Infect Control.

[5] Scott T, Mannion R, Marshall M, Davies H (2003). Does organisational culture influence health care performance? A review of the evidence. J Health Serv Res Policy.

[6] Palmieri PA, Peterson LT, Savage GT, Fottler MD (2009). Attribution theory and healthcare culture: Translational management science contributes a framework to identify the etiology of punitive clinical environments. Biennial Review of Health Care Management: Meso Perspective.

[7] Clark G (2002). Organisational culture and safety: an interdependent relationship. Aust Health Rev.

[8] Hellings J, Schrooten W, Klazinga NS, Vleugels A (2010). Improving patient safety culture. Int J Health Care Qual Assur.

[9] Noort MC, Reader TW, Shorrock S, Kirwan B (2016). The relationship between national culture and safety culture: implications for international safety culture assessments. J Occup Organ Psychol.

[10] Singer S, Falwell A, Gaba DM, Meterko M, Rosen A, Hartmann CW, Baker L (2009). Identifying organizational cultures that promote patient safety. Health Care Manag Rev.

[11] El-Jardali F, Dimassi H, Jamal D, Jaafar M, Hemadeh N (2011). Predictors and outcomes of patient safety culture in hospitals. BMC Health Serv Res.

[12] Mardon RE, Khanna K, Sorra J, Dyer N, Famolaro T (2010). Exploring relationships between hospital patient safety culture and adverse events. J Patient Saf.

[13] Palmieri PA, Peterson LT, Pesta BJ, Flit MA, Saettone DM, Savage GT, Khatri N, Fottler MD (2010). Safety culture as a contemporary healthcare construct: Theoretical review, research assessment, and translation to human resource management. Strategic Human Resource Management in Health Care.

[14] Shortell SM, Denise M, Rouseau DM, Gillies RR, Devers KJ, Simons TL (1991). Organizational assessment in intensive care units (ICUs): construct development, reliability, and validity of the ICU nurse-physician questionnaire. Med Care.

[15] Hofoss D, Deilkas E (2008). Roadmap for patient safety research: approaches and roadforks. Scandanavian J Public Health.

[16] World Alliance for Patient Safety (2008). Forward Programme: 2008–2009.

[17] Drösler SE, Klazinga NS, Romano PS, Tancredi DJ, Gogorcena Aoiz MA, Hewitt MC, Scobie S, Soop M, Wen E, Quan H (2009). Application of patient safety indicators internationally: a pilot study among seven countries. Int J Qual Health Care.

[18] Kruk ME, Freedman LP (2008). Assessing health system performance in developing countries: a review of the literature. Health Policy.

[19] Marcel JP, Alfa M, Baquero F, Etienne J, Goossens H, Harbarth S, Hryniewicz W, Jarvis W, Kaku M, Leclercq R (2008). Healthcare-associated infections: think globally, act locally. Clin Microbiol Infect.

[20] Hernandez K, Ramos E, Seas C, Henostroza G, Gotuzzo E (2005). Incidence of and risk factors for surgical-site infections in a Peruvian hospital. Inf Contrl Hosp Epidemiol.

[21] Velasco E, Thuler LC, Martins CA, Dias LM, Goncalves VM (1997). Nosocomial infections in an oncology intensive care unit. Am J Infect Control.

[22] Basu S, Andrews J, Kishore S, Panjabi R, Stuckler D (2012). Comparative performance of private and public healthcare systems in low- and middle-income countries: a systematic review. PLoS Med.

[23] Ramirez-Wong FM, Atencio-Espinoza T, Rosenthal VD, Ramirez E, Torres-Zegarra SL, Díaz Tavera ZR, Sarmiento López F, Silva Astete N, Campos Guevara F, Bazan Mendoza C (2015). Surgical site infections rates in more than 13,000 surgical procedures in three cities in Peru: findings of the international nosocomial infection control consortium. Surg Infect.

[24] Arrieta A, Suárez G, Hakim G (2018). Assessment of patient safety culture in private and public hospitals in Peru. Int J Qual Health Care.

[25] Payne SC, Bergman ME, Beus JM, Rodríguez JM, Henning JB (2009). Safety climate: leading or lagging indicator of safety outcomes?. J Loss Prev Process Ind.

[26] Colla JB, Bracken AC, Kinney LM, Weeks WB (2005). Measuring patient safety climate: a review of surveys. Qual Saf Health Care.

[27] Blegen MA, Gearhart S, O'Brien R, Sehgal NL, Alldredge BK (2009). AHRQ's hospital survey on patient safety culture: psychometric analyses. J Patient Saf.

[28] Nieva VF, Sorra J (2003). Safety culture assessment: A tool for improving patient safety in healthcare organizations. Qual Saf Health Care.

[29] Sorra J, Dyer N (2010). Multilevel psychometric properties of the AHRQ hospital survey on patient safety culture. BMC Health Serv Res.

[30] Chen IC, Li H-H (2010). Measuring patient safety culture in Taiwan using the hospital survey on patient safety culture (HSOPSC). BMC Health Serv Res.

[31] El-Jardali F, Jaafar M, Dimassi H, Jamal D, Hamdan R (2010). The current state of patient safety culture in Lebanese hospitals: a study at baseline. Int J Qual Health Care.

[32] Pfeiffer Y, Manser T (2010). Development of the German version of the hospital survey on patient safety culture: dimensionality and psychometric properties. Saf Sci.

[33] Sunol R, Vallejo P, Groene O, Escaramis G, Thompson A, Kutryba B, Garel P (2009). Implementation of patient safety strategies in European hospitals. Qual Saf Health Care.

[34] Güneş ÜY, Gürlek Ö, Sönmez M (2016). A survey of the patient safety culture of hospital nurses in Turkey. Collegian.

[35] Raeissi P, Reisi N, Nasiripour AA (2018). Assessment of patient safety culture in Iranian academic hospitals: strengths and weaknesses. J Patient Saf.

[36] Granel N, M-DJ M, Barth A, Papp K, Bernabeu-Tamayo MD (2019). Patient safety culture in Hungarian hospitals. Int J Health Care Qual Assur.

[37] Agency for Healthcare Research and Quality (2009). Cuestionario sobre la de seguridad de los pacientes en los hospitales.

[38] Fajardo-Dolci G, Rodriguez-Suarez J, Arboleya-Casanova H, Rojano-Fernandez C, Hernandez-Torres F, Santacruz-Varela J (2010). Cultura sobre seguridad del paciente en profesionales de la salud. Cirugía y Cirujanos.

[39] Ramírez-Martínez ME, González Pedraza-Avilés A (2017). Cultura de seguridad y eventos adversos en una clínica de primer nivel. Enfermería Universitaria.

[40] Gómez Ramírez O, Arenas Gutiérrez W, González Vega L, Garzón Salamanca J, Mateus Galeano E, Soto Gámez A (2011). Cultura de seguridad del paciente por personal de enfermeria en Bogata, Colombia. Ciencia y enfermería.

[41] Saturno Hernández PJ, Da Silva Gama ZA, de Oliveira Sousa SL, YA YAF, De Souza Oliveira AC (2007). Análisis de la cultura sobre seguridad del paciente en el ámbito hospitalario del Sistema Nacional de Salud español.

[42] Pinheiro MdP, Junior OCdS: Evaluación de la cultura de seguridad del paciente en una organización hospitalaria de un hospital universitario**.** Enfermería Global 2016, 16**:**309–324.

[43] Agency for Healthcare Research and Quality. Translation guidelines for the surveys on patient safety culture. Rockville; 2010.

[44] Flin R, Burns C, Mearns K, Yule S, Robertson EM (2006). Measuring safety climate in health care. Qual Saf Health Care.

[45] Hutchinson A, Cooper KL, Dean JE, McIntosh A, Patterson M, Stride CB, Laurence BE, Smith CM (2006). Use of a safety climate questionnaire in UK health care: factor structure, reliability and usability. Qual Saf Health Care.

[46] Fujishiro K, Gong F, Baron S, Jacobson CJ, DeLaney S, Flynn M, Eggerth DE. Translating questionnaire items for a multi-lingual worker population: the iterative process of translation and cognitive interviews with English-, Spanish-, and Chinese-speaking workers. Am J Ind Med. 2010;53:194–203. 10.1002/ajim.20733. 10.1002/ajim.20733 19650081

[47] Levin K, Willis GB, Forsyth BH, Norberg A, Kudela MS, Stark D, Thompson FE (2009). Using cognitive interviews to evaluate the Spanish-language translation of a dietary questionnaire. Surv Res Methods.

[48] Lopez GI, Figueroa M, Connor SE, Maliski SL (2008). Translation barriers in conducting qualitative research with Spanish speakers. Qual Health Res.

[49] Usunier J-C (2011). Language as a resource to assess cross-cultural equivalence in quantitative management research. J World Bus.

[50] Flaherty JA, Gaviria FM, Pathak D, Mitchell T, Wintrob R, Richman JA, Birz S (1988). Developing instruments for cross-cultural psychiatric research. J Nerv Ment Dis.

[51] van de Vijver FJR, Poortinga YH (1997). Towards an integrated analysis of bias in cross-cultural assessment. Eur J Psychol Assess.

[52] Waltz CF, Strickland OL, Lenz ER (2010). Measurement in nursing and health research.

[53] Knafl K, Deatrick J, Gallo A, Holcombe G, Bakitas M, Dixon J, Grey M (2007). The analysis and interpretation of cognitive interviews for instrument development. Res Nurs Health.

[54] Thrasher JF, Quah ACK, Dominick G, Borland R, Driezen P, Awang R, Omar M, Hosking W, Sirirassamee B, Boado M (2011). Using cognitive interviewing and behavioral coding to determine measurement equivalence across linguistic and cultural groups: an example from the international tobacco control policy evaluation project. Field Methods.

[55] Squires A (2009). Methodological challenges in cross-language qualitative research: a research review. Int J Nurs Stud.

[56] Maneesriwongul W, Dixon JK (2004). Instrument translation process: a methods review. J Adv Nurs.

[57] Willis GB (2005). Cognitive interviewing: a tool for improving questionnaire design.

[58] Erkut S (2010). Developing multiple language versions of instruments for intercultural research. Child Dev Perspect.

[59] Mason TC (2005). Cross-cultural instrument translation: assessment, translation, and statistical applications. Am Ann Deaf.

[60] Temple B (2005). Nice and tidy: translation and representation. Sociol Res Online.

[61] Acquadro C, Conway K, Hareendran A, Aaronson N (2008). Literature review of methods to translate health-related quality of life questionnaires for use in multinational clinical trials. Value Health.

[62] Wild D, Grove A, Martin M, Eremenco S, McElroy S, Verjee-Lorenz A, Erikson P (2005). Principles of good practice for the translation and cultural adaptation process for patient-reported outcomes (PRO) measures: report of the ISPOR task force for translation and cultural adaptation. Value Health.

[63] Squires A, Aiken LH, van den Heede K, Sermeus W, Bruyneel L, Lindqvist R, Schoonhoven L, Stromseng I, Busse R, Brzostek T (2013). A systematic survey instrument translation process for multi-country, comparative health workforce studies. Int J Nurs Stud.

[64] Beatty PC, Willis GB (2007). Research synthesis: the practice of cognitive interviewing. Public Opin Q.

[65] Weech-Maldonado R, Morales LS, Spritzer K, Elliott M, Hays RD (2001). Racial and ethnic differences in parents' assessments of pediatric care in Medicaid managed care. Health Serv Res.

[66] Garcia AA (2011). Cognitive interviews to test and refine questionnaires. Public Health Nurs.

[67] Harkness JA, Van de Vijver FJR, Mohler P (2003). Cross-cultural survey methods.

[68] Agency for Healthcare Research and Quality. Hospital Survey on Patient Safety Culture: Background and information for translators. Rockville: Agency for Healthcare Research and Quality; 2009.

[69] Agency for Healthcare Research and Quality (2004). Pilot study: Validity and reliability of the hospital survey on patient safety.

[70] Gjersing L, Caplehorn JR, Clausen T (2010). Cross-cultural adaptation of research instruments: language, setting, time and statistical considerations. BMC Med Res Methodol.

[71] Sousa VD, Rojjanasrirat W (2011). Translation, adaptation and validation of instruments or scales for use in cross-cultural health care research: a clear and user-friendly guideline. J Eval Clin Pract.

[72] Guillemin F, Bombardier C, Beaton D (1993). Cross-cultural adaptation of health-related quality of life measures: literature review and proposed guidelines. J Clin Epidemiol.

[73] Squires A (2008). Language barriers and qualitative nursing research: methodological considerations. Int Nurs Rev.

[74] Grant JS, Davis LL (1997). Selection and use of content experts for instrument development. Res Nurs Health.

[75] Bracken BA, Barona A (1991). State of the art procedures for translating, validating and using psychoeducational tests in cross-cultural assessment. Sch Psychol Int.

[76] Brislin RW (1970). Back-translation for cross-cultural research. J Cross-Cult Psychol.

[77] Chapman DW, Carter JF (1979). Translation procedures for the cross cultural use of measurement instruments. Educ Eval Policy Anal.

[78] Jones PS, Lee JW, Phillips LR, Zhang XE, Jaceldo KB (2001). An adaptation of Brislin’s translation model for cross-cultural research. Nurs Res.

[79] Chang AM, Chau JPC, Holroyd E (1999). Translation of questionnaires and issues of equivalence. J Adv Nurs.

[80] Tourangeau R, Jabine TB, Straf ML, Tanur JM, Tourangeau R (1984). Cognitive sciences and survey methods. Cognitive aspects of survey methodology: Building a bridge between disciplines.

[81] Miller K (2003). Conducting cognitive interviews to understand question-response limitations. Am J Health Behav.

[82] Polit DF, Beck CT (2006). The content validity index: are you sure you know what's being reported? Critique and recommendations. Res Nurs Health.

[83] Polit DF, Beck CT, Owen SV (2007). Is the CVI an acceptable indicator of content validity? Appraisal and recommendations. Res Nurs Health.

[84] Rubio DM, Berg-Weger M, Tebb SS, Lee ES, Rauch S (2003). Objectifying content validity: conducting a content validity study in social work research. Soc Work Res.

[85] Lawshe CH (1975). A quantitative approach to content validity. Pers Psychol.

[86] Lynn MR (1986). Determination and quantification of content validity. Nurs Res.

[87] Zamanzadeh V, Rassouli M, Abbaszadeh A, Alavi Majd H, Nikanfar A, Ghahramanian A (2015). Details of content validity and objectifying it in instrument development. Nurs Pract Today.

[88] Okochi J, Takahashi T, Takamuku K, Matsuda S, Takagi Y (2005). Reliability of a geriatric assessment instrument with illustrations. Geriatr Gerontol Int.

[89] Gorelick MH, Wagner D, McLellan SL (2008). Development and validation of a self-administered questionnaire to measure water exposures in children. Ambul Pediatr.

[90] Dufour S, Barkema HW, DesCôteaux L, DeVries TJ, Dohoo IR, Reyher K, Roy J-P, Scholl DT (2010). Development and validation of a bilingual questionnaire for measuring udder health related management practices on dairy farms. Prev Vet Med.

[91] Cicchetti DV, Sparrow SA (1981). Developing criteria for establishing interrater reliability of specific items: applications to assessment of adaptive behavior. Am J Ment Defic.

[92] Fleiss JL (1971). Measuring nominal scale agreement among many raters. Psychol Bull.

[93] Landis JR, Koch GG (1977). An application of hierarchical kappa-type statistics in the assessment of majority agreement among multiple observers. Biometrics.

[94] DeVillis RF. Scale development: theory and application. Thousand Oaks: Sage Publications; 2003.

[95] Napoles-Springer AM, Santoyo-Olsson J, O'Brien H, Stewart AL (2006). Using cognitive interviews to develop surveys in diverse populations. Med Care.

[96] Banville D, Desrosiers P, Genet-Volet Y (2000). Translating questionnaires and inventories using a cross-cultural translation technique. J Teach Phys Educ.

[97] Messick S (1966). The psychology of acquiescence: an interpretation of research evidence. ETS Res Bull Ser.

[98] Ray JJ (1983). Reviving the problem of acquiescent response bias. J Soc Psychol.

[99] Lavrakas PJ, editor. Encyclopedia of survey research methods. Thousand Oaks: SAGE Publications; 2008.

[100] Solís-Salazar M (2015). The dilemma of combining positive and negative items in scales. Psicothema.

