# Supplementary material for: Hospital survey on patient safety culture (HSOPSC): a multi-method approach for target-language instrument translation, adaptation, and validation to improve the equivalence of meaning for cross-cultural research
Source: BMC Nurs. 2020 Apr 13;19:23. doi: 10.1186/s12912-020-00419-9 (PMC7153229; doi:10.1186/s12912-020-00419-9)
Supplement: Supplementary file 1 — Additional file 1 Table S1. Item Comparison Across Instruments: Original English and Spanish Versions [file 12912_2020_419_MOESM1_ESM.docx]

Supplemental Table 1. Item Comparison Across Instruments: Original English and Spanish Versions

| **Item** | **Dim** | **AHRQ: HSOPSC ^1^  English Version (USA)** | **AHRQ: HSOPSC ^2^ Spanish Version (USA)** | **CUSEP: HSOPSC ^3^**  **Spanish Version (Spain)** | **STUDY: HSOPSC Initial Translation (T-Initial)*** | **STUDY: HSOPSC Final Version (T-Final)**** |
| --- | --- | --- | --- | --- | --- | --- |
| **A-1** | **1** | People support one another in this unit. | La gente se apoya una a la otra en esta unidad. | El personal se apoya mutuamente. | La gente se apoya mutuamente en esta unidad. | La gente se apoya el uno al otro en este servicio/ unidad. |
| **A-2** | **10** | We have enough staff to handle the workload. | Tenemos suficiente personal para hacer todo el trabajo. | Hay suficiente personal para afrontar la carga de trabajo. | Tenemos suficiente personal para manejar la carga de trabajo. | Tenemos suficiente personal para afrontar la carga de trabajo. |
| **A-3** | **1** | When a lot of work needs to be done quickly, we work together as a team to get the work done. | Cuando se tiene que hacer mucho trabajo rápidamente, trabajamos en equipo de trabajo para terminarlo. | Cuando tenemos mucho trabajo, colaboramos todos como un equipo para poder terminarlo. | Cuando se necesita hacer mucho trabajo rápidamente, trabajamos juntos, como un equipo, para conseguirlo. | Cuando se necesita hacer mucho trabajo rápidamente, colaboramos todos como un equipo para poder terminarlo. |
| **A-4** | **1** | In this unit, people treat each other with respect. | En esta unidad, el personal se trata con respeto. | En esta unidad nos tratamos todos con respeto. | En esta unidad, la gente se trata con respeto. | En este servicio/unidad, el personal se trata con respeto. |
| **A-5** | **10** | Staff in this unit work longer hours than is best for patient care. | El personal en esta unidad trabaja más horas de lo que es mejor para el cuidado del paciente. | A veces, no se puede proporcionar la mejor atención al paciente porque la jornada laboral es agotadora. | El personal en esta unidad trabaja más horas de lo conveniente para el cuidado del paciente. | El personal en este servicio/unidad trabaja más horas de lo que es mejor para el cuidado del paciente. |
| **A-6** | **3** | We are actively doing things to improve patient safety. | Estamos haciendo cosas de manera activa para mejorar la seguridad del paciente. | Tenemos actividades dirigidas a mejorar la seguridad del paciente. | Hacemos cosas de manera activa para mejorar la seguridad del paciente. | Hacemos actividades para mejorar la seguridad del paciente. |
| **A-7** | **10** | We use more agency/temporary staff than is best for patient care. | Usamos más personal de agencia/temporal de lo que es lo mejor para el cuidado del paciente. | En ocasiones no se presta la mejor atención al paciente porque hay demasiados sustitutos o personal temporal. | Usamos más personal temporal o de agencia de lo conveniente para el cuidado del paciente. | Usamos más personal de reemplazo o temporal de lo que es mejor para el cuidado del paciente. |
| **A-8** | **12** | Staff feel like their mistakes are held against them. | El personal siente que sus errores son considerados en su contra. | Si los compañeros o los superiores se enteran de que has cometido algún error, lo utilizan en tu contra. | El personal siente que sus errores juegan en su contra. | El personal siente que sus errores son considerados en su contra. |
| **A-9** | **3** | Mistakes have led to positive changes here. | Los errores han llevado a cambios positivos aquí. | Cuando se detecta algún fallo en la atención al paciente se llevan a cabo las medidas apropiadas para evitar que ocurra de nuevo. | Aquí, los errores han llevado a cambios positivos. | Los errores han llevado a realizar cambios positivos aquí. |
| **A-10** | **5** | It is just by chance that more serious mistakes don’t happen around here. | Es sólo por casualidad que errores más serios no ocurren aquí. | No se producen más fallos por casualidad. | Aquí no suceden errores más serios sólo por casualidad. | Es sólo por casualidad que errores más serios no suceden aquí. |
| **A-11** | **1** | When one area in this unit gets really busy, others help out. | Cuando un área en esta unidad está realmente ocupada, otras le ayudan. | Cuando alguien está sobrecargado de trabajo, suele encontrar ayuda en los compañeros. | Cuando un área en esta unidad se encuentra realmente ocupada, otras la ayudan. | Cuando el personal en este servicio/unidad está realmente ocupado, otros le ayudan. |
| **A-12** | **12** | When an event is reported, it feels like the person is being written up, not the problem. | Cuando se informa de un incidente, se siente que la persona está siendo reportada y no el problema. | Cuando se detecta algún fallo, antes de buscar la causa, buscan un “culpable”. | Cuando se reporta un evento, se tiene la sensación de que se reporta a la persona y no al evento. | Cuando se reporta un evento, se tiene la sensación de que se reporta a la persona y no al evento. |
| **A-13** | **3** | After we make changes to improve patient safety, we evaluate their effectiveness. | Después de hacer los cambios para mejorar la seguridad de los pacientes, evaluamos su efectividad. | Los cambios que hacemos para mejorar la seguridad del paciente se evalúan para comprobar su efectividad. | Después de hacer cambios para mejorar la seguridad del paciente, evaluamos su efectividad. | Después de hacer los cambios para mejorar la seguridad de los pacientes, evaluamos si los resultados son los esperados. |
| **A-14** | **10** | We work in "crisis mode" trying to do too much, too quickly. | Frecuentemente, trabajamos en "tipo crisis" intentando hacer mucho, muy rápidamente. | Trabajamos bajo presión para realizar demasiadas cosas demasiado deprisa. | Trabajamos en “modo de crisis”, tratando de hacer demasiado y muy rápidamente. | 14. Trabajamos a alta presión intentando realizar demasiadas cosas muy rápidamente. |
| **A-15** | **5** | Patient safety is never sacrificed to get more work done. | La seguridad del paciente nunca se sacrifica por hacer más trabajo. | Nunca se aumenta el ritmo de trabajo si eso implica sacrificar la seguridad del paciente. | La seguridad del paciente nunca es sacrificada en aras de tener más trabajo terminado. | La seguridad del paciente nunca se sacrifica por hacer más trabajo. |
| **A-16** | **12** | Staff worry that mistakes they make are kept in their personnel file. | El personal se preocupa de que los errores que cometen sean guardados en sus expedientes. | Cuando se comete un error, el personal teme que eso quede en su expediente. | Al personal le preocupaba que los errores cometidos permanezcan en sus expedientes personales. | 16. Cuando se comete un error, el personal teme que eso quede en su expediente. |
| **A-17** | **5** | We have patient safety problems in this unit. | Tenemos problemas con la seguridad de los pacientes en esta unidad. | En esta unidad hay problemas relacionados con la “seguridad del paciente”. | Tenemos problemas con la seguridad de los pacientes en esta unidad. | Tenemos problemas con la seguridad de los pacientes en este servicio/unidad. |
| **A-18** | **5** | Our procedures and systems are good at preventing errors from happening. | Nuestros procedimientos y sistemas son efectivos para la prevención de errores que puedan ocurrir. | Nuestros procedimientos y medios de trabajo son buenos para evitar errores en la asistencia. | Nuestros procedimientos y sistemas son buenos en la prevención de errores. | Nuestros procedimientos y procesos son efectivos para la prevención de errores que puedan ocurrir. |
| **B-1** | **2** | My supervisor/manager says a good word when he/she sees a job done according to established patient safety procedures. | Mi supervisor/director hace comentarios favorables cuando él/ella ve un trabajo hecho de conformidad con los procedimientos establecidos de seguridad de los pacientes. | Mi superior/jefe expresa su satisfacción cuando intentamos evitar riesgos en la seguridad del paciente. | Mi gerente hace comentarios favorables cuando ve un trabajo hecho de acuerdo a los procedimientos establecidos de seguridad del paciente. | Mi jefe hace comentarios favorables cuando ve un trabajo hecho de acuerdo con los procedimientos establecidos de seguridad del paciente. |
| **B-2** | **2** | My supervisor/manager seriously considers staff suggestions for improving patient safety. | Mi supervisor/director considera seriamente las sugerencias del personal para mejorar la seguridad de los pacientes. | Mi superior/jefe tiene en cuenta, seriamente, las sugerencias que le hace el personal para mejorar la seguridad del paciente. | Mi gerente considera seriamente las sugerencias del personal a fin de mejorar la seguridad del paciente. | Mi jefe considera seriamente las sugerencias del personal para mejorar la seguridad de los pacientes. |
| **B-3** | **2** | Whenever pressure builds up, my supervisor/ manager wants us to work faster, even if it means taking shortcuts. | Cuando la presión se incrementa, mi supervisor/director quiere que trabajemos más rápido, aún si esto significa simplificar las labores del trabajo. | Cuando aumenta la presión del trabajo, mi superior/jefe pretende que trabajemos más rápido, aunque se pueda poner en riesgo la seguridad del paciente. | Siempre que la presión aumenta, mi supervisor/ gerente quiere que trabajemos más rápido, incluso si esto significa simplificar el trabajo. | Cuando la presión se incrementa, mi jefe quiere que trabajemos más rápido, aún si esto significa acortar en forma negativa los procesos. |
| **B-4** | **2** | My supervisor/manager overlooks patient safety problems that happen over and over. | Mi supervisor/director no hace caso de los problemas de seguridad en los pacientes que ocurren una y otra vez. | Mi superior/jefe pasa por alto los problemas de seguridad del paciente que ocurren habitualmente. | Mi gerente pasa por alto los problemas en la Seguridad del paciente que ocurren una y otra vez. | Mi jefe no hace caso de los problemas de seguridad en los pacientes que ocurren una y otra vez. |
| **C-1** | **6** | We are given feedback about changes put into place based on event reports. | La Dirección nos informa sobre los cambios realizados que se basan en informes de incidentes. | Cuando notificamos algún incidente, nos informan sobre qué tipo de actuaciones se han llevado a cabo. | Se nos informa sobre los cambios hechos en base a reportes de eventos. | Se nos informa sobre los cambios hechos en base a reportes de eventos. |
| **C-2** | **7** | Staff will freely speak up if they see something that may negatively affect patient care. | El personal habla libremente si ve algo que podría afectar negativamente el cuidado del paciente. | Cuando el personal ve algo que puede afectar negativamente a la atención que recibe el paciente, habla de ello con total libertad. | El personal habla libremente si ve algo que podría afectar negativamente el cuidado del paciente. | El personal habla libremente si ve algo que podría afectar negativamente el cuidado del paciente. |
| **C-3** | **6** | We are informed about errors that happen in this unit. | Se nos informa sobre los errores que se cometen en esta unidad. | Se nos informa de los errores que ocurren en este servicio/unidad. | Se nos informa sobre los errores que ocurren en esta unidad. | Se nos informa sobre los errores que se cometen en este servicio/unidad. |
| **C-4** | **7** | Staff feel free to question the decisions or actions of those with more authority. | El personal se siente libre de cuestionar las decisiones o acciones de aquellos con mayor autoridad. | El personal puede cuestionar con total libertad las decisiones o acciones de sus superiores. | El personal se siente libre de cuestionar las decisiones o acciones de las personas con más autoridad. | El personal se siente libre de cuestionar las decisiones o acciones de aquellos con mayor autoridad. |
| **C-5** | **6** | In this unit, we discuss ways to prevent errors from happening again. | En esta unidad, hablamos sobre formas de prevenir los errores para que no se vuelvan a cometer. | En mi servicio/unidad discutimos de qué manera se puede evitar que un error vuelva a ocurrir. | En esta unidad debatimos maneras de prevenir errores para que no ocurran nuevamente. | En este servicio/unidad debatimos maneras de prevenir errores para que no ocurran nuevamente. |
| **C-6** | **7** | Staff are afraid to ask questions when something does not seem right. | El personal tiene miedo de hacer preguntas cuando algo no parece estar bien. | El personal teme hacer preguntas sobre lo que parece que se ha hecho de forma incorrecta. | El personal teme hacer preguntas cuando algo no parece estar bien. | El personal tiene miedo de hacer preguntas cuando algo no parece estar bien. |
| **D-1** | **8** | When a mistake is made, but is caught and corrected before affecting the patient, how often is this reported? | Cuando se comete un error, pero es descubierto y corregido antes de afectar al paciente, ¿qué tan a menudo es reportado? | Se notifican los errores que son descubiertos y corregidos antes de afectar al paciente. | Cuando se comete un error, pero es descubierto y corregido antes de afectar al paciente, ¿con qué frecuencia es reportado? | Cuando se comete un error, pero es descubierto y corregido antes de afectar al paciente, ¿con qué frecuencia es reportado? |
| **D-2** | **8** | When a mistake is made, but has no potential to harm the patient, how often is this reported? | Cuando se comete un error, pero no tiene el potencial de dañar al paciente, ¿qué tan frecuentemente es reportado? | Se notifican los errores que previsiblemente no van a dañar al paciente. | Cuando se comete un error, pero no es potencialmente dañino para el paciente, ¿con qué frecuencia es reportado? | Cuando se comete un error, pero no tiene el potencial de dañar al paciente, ¿qué tan frecuentemente es reportado? |
| **D-3** | **8** | When a mistake is made that could harm the patient, but does not, how often is this reported? | Cuando se comete un error que pudiese dañar al paciente, pero no lo hace, ¿qué tan a menudo es reportado? | Se notifican los errores que no han tenido consecuencias adversas, aunque previsiblemente podrían haber dañado al paciente. | Cuando se comete un error que podría dañar al paciente, pero no lo llega a hacer, ¿con qué frecuencia es reportado? | Cuando se comete un error que pudiese dañar al paciente, pero no lo hace, ¿qué tan a menudo es reportado? |
| **F-1** | **4** | Management in this facility provides a work climate that promotes patient safety. | La Dirección de este hospital provee de un ambiente laboral que promueve la seguridad del paciente. | La gerencia o la dirección del hospital facilita un clima laboral que favorece la seguridad del paciente. | La administración de este hospital provee un clima de trabajo que promueve la seguridad del paciente. | La dirección de este hospital/clínica provee de un clima o ambiente laboral que favorece la seguridad del paciente. |
| **F-2** | **9** | Units in this facility do not coordinate well with each other. | Las unidades de este hospital no se coordinan bien entre ellas. | Las diferentes unidades del hospital no se coordinan bien entre ellas. | Las unidades de este hospital no coordinan bien entre sí. | Las unidades de este hospital no coordinan bien entre sí. |
| **F-3** | **11** | Things “fall between the cracks” when transferring patients from one unit to another. | La información de los pacientes se pierde cuando éstos se transfieren de una unidad a otra. | La información de los pacientes se pierde, en parte, cuando éstos se transfieren desde una unidad/servicio a otra. | Las cosas se pierden cuando los pacientes son transferidos de una unidad a otra. | La información y/o objetos de los pacientes se pierde cuando éstos se transfieren de un servicio/unidad a otra. |
| **F-4** | **9** | There is good cooperation among units that need to work together. | Hay buena cooperación entre las unidades del hospital que necesitan trabajar juntas. | Hay una buena cooperación entre las unidades/servicios que tienen que trabajar conjuntamente. | Existe un buen grado de cooperación entre las unidades que necesitan trabajar juntas. | Hay buena cooperación entre las unidades del hospital/clínica que necesitan trabajar juntas. |
| **F-5** | **11** | Important patient care information is often lost during shift changes. | Se pierde a menudo información importante de cuidado de pacientes durante cambios de turno. | En los cambios de turno se pierde con frecuencia información importante sobre la atención que ha recibido el paciente. | Con frecuencia se pierde importante información sobre el cuidado del paciente durante los cambios de turno. | Se pierde a menudo información importante de cuidado de pacientes durante cambios de turno. |
| **F-6** | **9** | It is often unpleasant to work with staff from other units in this facility. | Frecuentemente es desagradable trabajar con personal de otras unidades en este hospital. | Suele resultar incómodo tener que trabajar con personal de otros servicios/unidades. | Frecuentemente es desagradable trabajar con personal de otras unidades en este hospital. | Frecuentemente es desagradable trabajar con personal de otras unidades en este hospital/clínica. |
| **F-7** | **11** | Problems often occur in the exchange of information across units in this facility. | A menudo surgen problemas en el intercambio de información a través de unidades de este hospital. | El intercambio de información entre los diferentes servicios es habitualmente problemático. | A menudo surgen problemas en el intercambio de información a través de las unidades en este hospital. | A menudo surgen problemas en el intercambio de información a través de las unidades en este hospital/clínica. |
| **F-8** | **4** | The actions of management in this facility show that patient safety is a top priority. | Las acciones de la Dirección de este hospital muestra que la seguridad del paciente es altamente prioritaria. | La gerencia o dirección del hospital muestra con hechos que la seguridad del paciente es una de sus prioridades. | Las acciones de la administración de este hospital muestran que la seguridad del paciente es una prioridad máxima. | Las acciones de la dirección de este hospital/clínica muestra que la seguridad del paciente es altamente prioritaria. |
| **F-9** | **4** | Management in this facility seems interested in patient safety only after an adverse event happens. | La Dirección del hospital parece interesada en la seguridad del paciente sólo después de que ocurre un incidente adverso. | La gerencia/dirección del hospital sólo parece interesarse por la seguridad del paciente cuando ya ha ocurrido algún suceso adverso en un paciente. | La administración de este hospital parece interesarse en la seguridad del paciente sólo después de que ha ocurrido un evento adverso. | La dirección del hospital/clínica parece interesada en la seguridad del paciente sólo después de que ocurre un evento adverso. |
| **F-10** | **9** | Units in this facility work well together to provide the best care for patients. | Las unidades del hospital trabajan bien juntas para proveer el mejor cuidado para los pacientes. | Los servicios/unidades trabajan de forma coordinada entre sí para proporcionar la mejor atención posible. | Las unidades en este hospital trabajan bien juntas para proveer el mejor cuidado a los pacientes. | Los servicios/unidades en este hospital/clínica trabajan bien unidos para proveer el mejor cuidado a los pacientes. |
| **F-11** | **11** | Shift changes are problematic for patients in this facility. | Los cambios de turnos son problemáticos para los pacientes en este hospital. | Surgen problemas en la atención de los pacientes como consecuencia de los cambios de turno. | En este hospital los cambios de turno son problemáticos para los pacientes. | Los cambios de turnos generan problemas para los pacientes en este hospital/clínica. |

* The initial translation of the instrument (translation and expert review/revision) is provided as the initial time, or T-Initial.

** The final translation at the end of the study is provided as the final time, or T-Final. The two comparisons provide additional information

about the subtle changes across the study.

Links to access the instruments described in this supplemental table:

1. AHRQ Hospital Survey on Patient Safety Culture: English Version 1.0. Available at: https://www.ahrq.gov/sites/default/files/wysiwyg/sops/quality-patient-safety/patientsafetyculture/hospitalscanform.pdf
2. AHRQ Hospital Survey on Patient Safety Culture: Spanish Version 1.0. Available at: https://www.ahrq.gov/sites/default/files/wysiwyg/sops/surveys/hospital/hospital_survey-spanish.pdf
3. Cuestionario sobres seguridad de los pacientes: Versión española del Hospital Survey on Patient Safety. Available at: https://www.mscbs.gob.es/organizacion/sns/planCalidadSNS/docs/CuestionarioSeguridadPacientes1.pdf
